# Regression of Left Ventricular Hypertrophy in Patients Combined with Peritoneal Dialysis and Hemodialysis

**DOI:** 10.1155/2022/2652380

**Published:** 2022-11-26

**Authors:** Gao Luyan, Zhang Haixia, Feng Sheng, Sun Gang, Zhu Jing, Lu Ying, Jiang Linsen, Song Kai, Wang Zhi, Shen Huaying

**Affiliations:** The Second Affiliated Hospital of Soochow University, Suzhou, China

## Abstract

**Methods:**

This retrospective study enrolled 58 patients at The Second Affiliated Hospital of Soochow University who switched from PD to PHD. Clinical data and echocardiographic examination results were collected. Data from the two groups with a normal distribution were compared with the paired *t*-test. A *p*value <0.05 (two-tailed) was considered statistically significant.

**Results:**

A total of 58 subjects were enrolled, including 46 males and 12 females, with a median age of 50.2 ± 11.1 (47–68) years. The mean duration of peritoneal dialysis was 67.2 ± 33.6 months. Before and after PHD, the ultrafiltration volume (*p* = 0.021) and hemoglobin (*p* = 0.001) were increased, while SBP (*p* = 0.002), DBP (*p* = 0.002), phosphorus (*p* < 0.001), and ESA dosage (*p* < 0.001) were decreased. Before and after combined dialysis (PHD), the incidence of LVH was 76.4% and 61.8%, respectively (*p* = 0.013), and LVMI decreased from 173.8 ± 86.2 g/m^2^ to 160.6 ± 78.5 g/m^2^ (*p* < 0.001).

**Conclusion:**

Compared with PD alone, the combination of PD and HD resulted in regression of LVH and reduced LVMI.

## 1. Introduction

Peritoneal dialysis is an effective treatment for uremia patients [1, 2]. In long-term PD patients, peritonitis recurrence, loss of residual renal function, and deterioration of peritoneal membrane function may cause ultrafiltration failure, fluid overload, and toxin accumulation, which may finally lead to technology failure and death [3–5]. Combining PD with hemodialysis (PHD) may be an effective solution for these patients [6, 7]. Several studies have already confirmed the benefits of PHD, including maintaining fluid balance, achieving dialysis adequacy, and prolonged life expectancy [8, 9].

It is well-established that cardiovascular disease is the primary cause of death in ESRD patients [10, 11]. Our previous studies have confirmed a high prevalence of left ventricular systolic and diastolic dysfunction, LVH, and valvular calcification in PD patients [12–14]. Many studies reported that hypertension, fluid overload, and phosphorus are risk factors for LVH in dialysis patients [15–17]. These risk factors can be alleviated after PHD, which may lead to the remission of LVH. We conducted this study to research cardiac structure change and function in patients before and after PHD.

## 2. Patients and Methods

**Figure 1:**
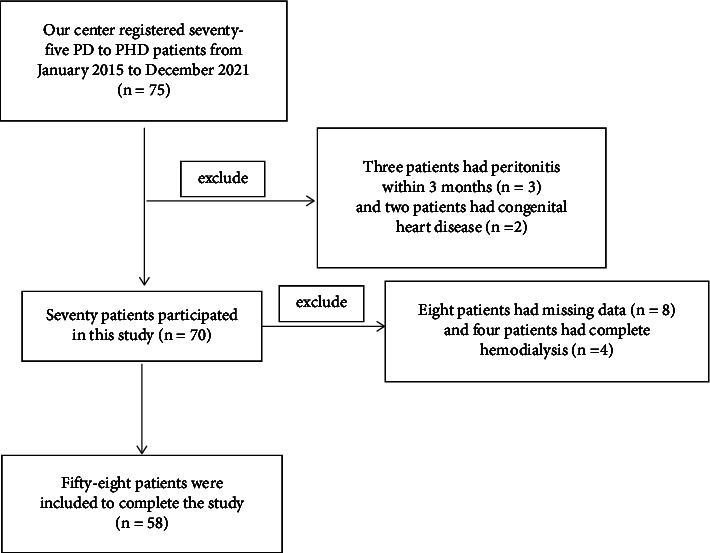
The experimental flow chart.

PHD was defined as patients receiving combined therapy of peritoneal dialysis and hemodialysis [8]. In this retrospective study, there were 75 patients enrolled at The Second Affiliated Hospital of Soochow University who switched from PD alone to combination therapy with PD and HD between Jan 1, 2015, and Dec 30, 2021. Reasons for the switch to PDH include dialysis inadequacy (*n* = 38), ultrafiltration failure (*n* = 25), and fluid overload (*n* = 12). Seventeen patients were excluded because of missing data (*n* = 8), transfer to HD (*n* = 4), peritonitis within three months (*n* = 3), and congenital heart disease (*n* = 2). Thus, only 58 patients participated in this study (Figure 1).

### 2.1. Indication of Combination Therapy with PD and HD

Thirty-eight patients received six days of PD and one session of HD per week. Kt/V of HD was targeted from 1.0 to 1.2. Other 20 patients received four days of PD and two sessions of HD per week.

Peritoneal dialysis was not conducted on the day of the HD session, defined as the day of peritoneal rest. For the HD prescription, no patient used a high-flux membrane dialyzer. Twelve patients received hemofiltration every two weeks, and nine received hemoperfusion once a month.

### 2.2. Physical and Laboratory Examinations

Blood pressure (BP) and body weight (BW) were measured before echocardiographic studies in these patients. The dose of erythropoietin stimulating agent (ESA) for one week was also analyzed. Clinical data, including age, gender, body mass index (BMI), dialysis vintage, history of diabetes, statins, ca channel blockers, renin-angiotensin system blockers, and *α β*-receptor blockers, as well as combination preparations, were collected from all patients. The blood test was performed just before the HD session on the dialysis day. We collected fasting biochemical blood indices from all patients, including serum creatinine, urea nitrogen, albumin (Alb), prealbumin (PA), PTH, serum Ca, serum P, CRP, triglyceride (TG), TC, high-density lipoprotein, and low-density lipoprotein levels.

### 2.3. Definition of Ultrafiltration Volume

We calculated the daily UF volume by averaging the total weekly UF volume. Weekly UF volume includes ultrafiltration volume of peritoneal dialysis and hemodialysis.

### 2.4. Echocardiographic Examination

Cardiac sonography was examined before the HD session. We calculate the LV mass according to the following equation:

(1) $$\begin{array}{c} \text{LV mass}\left(g\right)=0.8*1.04*\left[\left(\text{LVIDD}+\text{LVPWT}+\text{IVST}\right)3-\text{LVIDD}3\right]+0.6\text{,}\\ \text{LV mass index}=\text{LVMMSA}{\left(\frac{g}{m^{2}}\right)}^{1.3}\text{.} \end{array}$$

LVH was defined as the LV mass/height 2.7 (LV mass divided by height in meters in the power of 2.7) >52 g/m^2^. 7 in men and >47 g/m^2^. 7 in women as suggested by the 2013 ESH/ESC guidelines [10]. LV systolic function was assessed by ejection fraction (EF) measurement, and systolic dysfunction was defined as an EF <50%. Results of two echocardiographic data were collected at the initiation of PHD and during the following time. All echocardiographic measurements were performed by experienced technicians blinded to the clinical conditions.

### 2.5. Statistical Analysis

Data were expressed as mean ± SD or median (interquartile range) based on the distribution type. The statistical analysis was performed using SPSS 24.0 (IBM SPSS, Somers, N.Y., USA). Two groups of data with a normal distribution were compared with the paired *t*-test, skewed data were compared with the Mann–Whitney *U* test, and categorical data were compared with the *χ*2 test. Univariate logistic regression analysis was performed to estimate the relationship with LVMI improvement. Factors enrolled in the multivariate regression analysis were based on the clinical significance or univariate logistic regression results (factors with *p* < 0.1). Thus, dialysis vintage, SBP, DBP, HGB, and ultrafiltration volume were enrolled in multivariate regression analysis. A *p* value <0.05 (two-tailed) was considered statistically significant.

## 3. Results

A total of 58 subjects were enrolled, including 46 males and 12 females, with a median age of 50.2 ± 11.1 (47–68) years. The mean duration of peritoneal dialysis was 67.2 ± 33.6 months. All 58 patients were on CAPD before transfer to PHD. The follow-up of combined dialysis (PHD) was 12.2 ± 2.4 months. The primary causes of chronic renal failure included 21 cases of chronic nephritis, 11 cases of hypertensive kidney, 5 cases of diabetic nephropathy, 2 cases of polycystic kidney, and 19 cases of other causes. All patients were treated with erythropoietin (EPO), 50% with RAAS receptor blockers, 53.4% with beta-blockers, and 75.9% with CCB (Table 1). The number of antihypertensives, including RAAS inhibitors, was unchanged during the observation period. There were twelve patients taking furosemide dosing from 60 mg/d to 200 mg/d. During the study, there was no heart failure, cardiovascular events, peritonitis, and death. Besides, there was no hospitalization during the study period.

**Figure 2:**
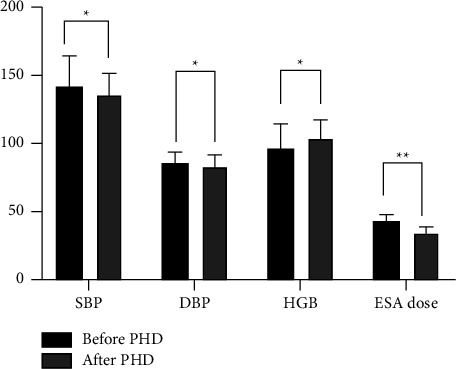
Comparison of SBP, DBP, hemoglobin, and ESA dosage before and after PHD (ESA dose, 1000 u/w; ^*∗*^*p* < 0.05; ^*∗∗*^*p* < 0.001).

Before and after PHD, the ultrafiltration volume (*p* = 0.021) and hemoglobin (*p* = 0.001) were increased, while SBP (*p* = 0.002), DBP (*p* = 0.002), phosphorus (*p* < 0.001) and ESA dosage (*p* < 0.001) were decreased. Other laboratory parameters, including Scr, BUN, ALB, iPTH, and calcium, did not reach statistical differences (Table 2, Figure 2). After PHD, 18 subjects (30%) reduced the proportion of 2.5% peritoneal dialyzate.

**Figure 3:**
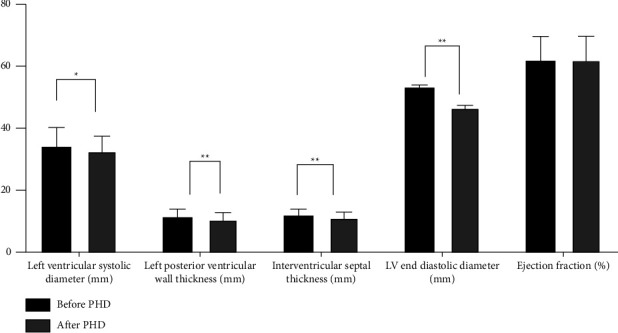
Comparison of echocardiographic results before and after PHD (^*∗*^*p* < 0.05; ^*∗∗*^*p* < 0.001).

**Figure 4:**
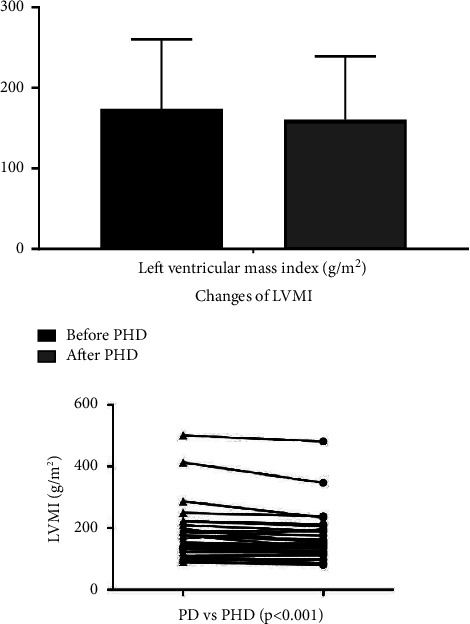
Comparison of left ventricular mass index results before and after PHD (*p* < 0.001).

This study showed that left ventricular systolic diameter (*p* = 0.002), left ventricular posterior wall thickness (*p* < 0.001), and interventricular septum thickness (*p* < 0.001) had significant differences during the follow-up period. Before and after combined dialysis (PHD), the incidence of LVH was 76.4% and 61.8%, respectively (*p* = 0.013). After PHD, 41 patients (75.6%) showed an improvement in LVMI. LVMI decreased from 173.8 ± 86.2 g/m^2^ to 160.6 ± 78.5 g/m^2^ (*p* < 0.001). At the same time, the EF value did not change significantly during the follow-up period (Table 3, Figures 3 and 4).

We performed the univariate and multivariate analysis of factors (after PHD) associated with LVMI improvement. In univariate analysis, we found that SPB (*p* = 0.021), DBP (*p* = 0.015), ultrafiltration volume (*p* = 0.005), hemoglobin (*p* = 0.023), and ESA dosage (*p* = 0.039) were associated with LVMI decrease. In multivariate analysis, there were only SPB (*p* = 0.014), DBP (*p* = 0.034), and ultrafiltration volume (*p* = 0.009) associated with LVMI improvement (Table 4).

## 4. Discussion

The effectiveness of PD in the Chinese population has already been proved by the “PD first policy” in Hong Kong [2]. However, in long-term PD patients, deterioration of peritoneal membrane function, dialysis inadequacy, and fluid overload are significant causes of technique failure and death [3, 4]. The lack of biocompatible dialyzate in mainland China and the limited use of automated peritoneal dialysis (APD) due to medical insurance policies may worsen these problems. In recent years, several studies have confirmed the effectiveness of PHD in these subjects. Based on the evidence above, patients who cannot continue PD alone switch to PHD in our center.

There were more male patients than female patients receiving PHD. This may cause more dialysis insufficiency in male than female PD patients. This result is also found in research conducted in Taiwan [18]. Primary nephritis is the most common cause in this study. However, a higher proportion (18.9%) of ESRD caused by hypertension was observed. One significant reason may be hypertensive nephropathy combined with paralleled cardiac disease, causing more strict volume control in long-term dialysis patients.

In this study, compared to PD alone, patients who received PHD showed better blood pressure control, increased ultrafiltration volume, decreased phosphorus, and elevated HGB with lower ESA usage. These findings are consistent with previous studies [19–21].

This study also reveals the amelioration of left ventricular hypertrophy and left ventricular diastolic function after receiving PHD treatment. There may be several reasons for the regression of LVH and reduced LVMI observed after PHD. Firstly, reduced fluid overload and better blood pressure control are the primary factors for this phenomenon [22]. Ozkahya et al. report that the volume decrease in dialysis patients can achieve reasonable long-term BP control and decreased LVMI [23]. Secondly, elevated HGB also affects reducing LVMI [24]. Furthermore, better phosphorus control is also a benefit for the decreased prevalence of LVH [16].

The present study's limitations include a lack of controls, a small number of patients, and a short follow-up period. Some factors, such as residual renal function, combined obstructive sleep apnea dosage, and CKD-MBD disorders, that may affect LVMI were lacking in this study. Further studies are needed to focus on these issues. We look forward to multicenter and large-scale prospective research in the future. In conclusion, the present study demonstrates that, compared with PD alone, PD and HD's combination resulted in regression of LVH and reduced LVMI.

## Figures and Tables

**Table 1:** Characteristics of patients.

Variables	Results
Age (years)	50.2 ± 11.5 (47～68)
Male, *n* (%)	46 (79.3)
Dialysis vintage (months)	(26～154)
Diabetes, *n* (%)	5 (8.6)
Drug	—
Diuretics, *n* (%)	15 (25.9)
RAS inhibitor, *n* (%)	29 (50)
CCB, *n* (%)	44 (75.9)
*α*-receptor antagonist, *n* (%)	20 (34.5)
*β*-receptor antagonist, *n* (%)	31 (53.4)
EPO, *n* (%)	58 (100)

CCB, Ca^2+^ channel blockers; RAS, renin-angiotensin system; EPO, erythropoietin.

**Table 2:** Comparison of clinical and laboratory parameters before and after PHD.

	Before PHD	After PHD	*p*
Body weight (kg)	61.1 ± 9.0	59.3 ± 8.6	0.453
SBP (mmHg)	141.7 ± 21.8	135.5 ± 15.6	0.002
DBP (mmHg)	85.7 ± 7.9	82.7 ± 8.3	0.002
Ultrafiltration volume (mL/d)	1012 ± 553	1233 ± 531	0.021
PD	1012 ± 553	668 ± 222	
HD	0	470 ± 150	
iPTH (pg/ml)	244 (126, 381)	225 (114, 354)	0.47
Hb (g/L)	96.0 ± 18.4	103.3 ± 14.0	0.001
ALB (g/L)	36.1 ± 5.2	36.4 ± 4.8	0.38
Ca (mmol/L)	2.32 ± 0.16	2.29 ± 0.16	0.20
P (mmol/L),	1.96 ± 0.54	1.84 ± 0.49	<0.001
BUN (mmol/L)	19.2 ± 5.5	18.7 ± 4.8	0.24
Scr (umol/L)	1209.6 ± 259.4	1227.8 ± 247.3	0.54
ESA dosage (10^4^ u/W)	4.3 ± 0.4	3.5 ± 0.3	<0.001

SBP, systolic blood pressure; DBP, diastolic blood pressure; iPTH, intact parathyroid hormone; Hb, hemoglobin; ALB, albumin; Ca, calcium; P, phosphorus; BUN, urea nitrogen; Scr, serum creatinine; ESA, erythropoietin stimulating agents.

**Table 3:** Comparison of echocardiographic results before and after PHD.

	Before PHD	After PHD	*p*
Aortic diameter (mm)	33.86 ± 4.91	33.76 ± 4.92	0.083
Left ventricular systolic diameter (mm)	34.11 ± 6.12	32.41 ± 5.15	0.002
Left posterior ventricular wall thickness (mm)	11.43 ± 2.61	10.68 ± 2.12	<0.001
Interventricular septal thickness (mm)	11.61 ± 2.29	10.88 ± 2.10	<0.001
LV end diastolic diameter (mm)	53.22 ± 0.61	46.83 ± 0.59	<0.001
Left ventricular mass index (g/m^2^)	173.8 ± 86.2	160.6 ± 78.5	<0.001
Left ventricular hypertrophy, *n* (%)	27 (76.4%)	21 (61.8%)	0.013
Pulmonary artery pressure (mmHg)	38.0 ± 6.55	37.8 ± 6.57	0.07
Ejection fraction (%)	61.91 ± 7.74	62.33 ± 7.79	0.32

**Table 4:** Univariate and multivariate analysis of factors (after PHD) associated with LVMI change.

Parameter	Univariate		Multivariate	
	*β*	*p*	*β*	*p*
Age (years)	0.034	NS	—	—
Dialysis vintage (years)	0.159	NS	0.122	NS
SBP (mmHg)	0.031	0.012	0.026	0.014
DBP (mmHg)	0.018	0.015	0.019	0.034
Ultrafiltration volume (mL/d)	0.010	0.005	0.012	0.009
iPTH (pg/ml)	0.160	NS	—	—
Hb (g/L)	0.32	0.023	0.171	0.054
ALB (g/L)	−0.112	NS	—	—
Ca (mmol/L)	0.24	NS	—	—
P (mmol/L)	0.26	NS	—	—
BUN (mmol/L)	0.30	NS	—	—
Scr (umol/L)	0.027	NS	—	—
ESA dosage (10^4^ u/W)	0.43	0.039	—	—

SBP, systolic blood pressure; DBP, diastolic blood pressure; iPTH, intact parathyroid hormone; Hb, hemoglobin; ALB, albumin; Ca, calcium; P, phosphorus; BUN, urea nitrogen; Scr, serum creatinine; ESA, erythropoietin stimulating agents.

## Data Availability

Available upon request.
